# Enhancing Sleep in NCAA Division I Athletes: A Study of Sleep Extension Feasibility

**DOI:** 10.1177/19417381261496986

**Published:** 2026-10-28

**Authors:** Joshua T. Goldman, Calvin Duffaut, Nicolas Hatamiya, Basil Ike, Brian Donohoe, Nelson F. Boland, Jeremy Vail, Jeongeun Kim, Kristen E. Holmes

**Affiliations:** Division of Sports Medicine, Department of Family Medicine, University of California, Los Angeles, California, and Department of Intercollegiate Athletics, University of California, Los Angeles, California; Division of Sports Medicine, Department of Family Medicine, University of California, Los Angeles, California, and Department of Intercollegiate Athletics, University of California, Los Angeles, California; Department of Orthopaedic Surgery and Department of Family and Community Medicine, University of California, San Francisco, California; Division of Sports Medicine, Department of Family Medicine, University of California, Irvine, California; Division of Sports Medicine, Department of Family Medicine, University of California, Los Angeles, California; Division of Sports Medicine, Department of Family Medicine, University of California, Los Angeles, California; Department of Intercollegiate Athletics, University of California, Los Angeles, California; Department of Performance Science and Thought Leadership, WHOOP, Inc, Boston, Massachusetts; Department of Performance Science and Thought Leadership, WHOOP, Inc, Boston, Massachusetts

**Keywords:** Sleep extension, sleep consistency, collegiate athletes, sleep duration, feasibility study

## Abstract

**Background:**

Sleep extension has been shown to improve varied aspects of athletic performance in collegiate athletes. Such studies have been conducted in small cohorts in controlled settings utilizing obsolete monitoring technology. The feasibility of sleep extension has not been evaluated in a broader cohort of collegiate athletes with modern monitoring technology.

**Hypothesis:**

Sleep extension may not be feasible in National Collegiate Athletic Association (NCAA) Division I athletes.

**Study Design:**

Crossover study.

**Level of Evidence:**

Level 3.

**Methods:**

Baseline sleep data were collected from NCAA Division I athletes (women’s soccer, men’s soccer, baseball, and football) during a 2-week period. Subjects were then instructed to extend their total sleep time to 10 hours per night for 2 weeks. All sleep baseline and extension data were collected in the offseason, remote from school examinations. WHOOP bands recorded all sleep metrics. Data were analyzed using paired-samples *t* tests and Wilcoxon signed rank tests.

**Results:**

A total of 50 subjects (14 female, 36 male) participated. The study compared total sleep time (hours) and sleep consistency (percent) - the stability of sleep-wake schedule over time, between baseline and sleep extension phases. Sample size varied for both metrics, ranging from 46 to 49 participants, respectively. There was a statistically significant increase in sleep consistency during the sleep extension phase (baseline, 60.09 ± 12.6%; extension, 64.42 ± 12.4%; *P* = 0.02). However, total sleep time showed no significant difference between the 2 phases (baseline, 7.63 ± 0.88 hours; extension, 7.51 ± 0.88 hours; *P* = 0.27).

**Conclusion:**

Extension efforts improved sleep consistency but did not significantly impact total sleep time suggesting that a sleep extension intervention can enhance sleep consistency in NCAA Division I athletes but does not significantly impact total sleep time. Sleep extension may be challenging for collegiate athletes given the time-intensive demands of both academics and sports. While sleep extension may be feasible in professional sports, it does not appear to be so in the college setting.

**Clinical Relevance:**

While the sleep extension intervention improved sleep consistency, it did not improve total sleep time, questioning the feasibility of sleep extension in collegiate sports.

Sleep is essential for the health and wellbeing of all people. Inadequate sleep has been associated with mood disturbances, fatigue, daytime sleepiness, and negative effects on athletic and academic performance.^1,3-5,7,8,11^ Research has demonstrated that collegiate student-athletes are not meeting current sleep guidelines.^12^ Current recommendations suggest that adults sleep for 7 to 9 hours and young adults strive for >9 hours.^13^ Goldman et al^4^ found the average sleep time of collegiate athletes to be only 6.8 hours. Other studies have shown that 25% to 30% of student-athletes sleep <6 hours a night.^5^ This has led organizations, including the National Collegiate Athletic Association (NCAA) and International Olympic Committee (IOC), to release consensus statements regarding sleep and its effects on mental and physical health in athletes.^7,14^

Several studies have shown that sleep extension, defined here as increasing the time spent in bed and/or asleep, can improve various performance metrics in athletes. Mah et al^8^ found significant improvements in free throw percentage and 3-point field goal percentage in 11 collegiate basketball players after sleep extension. Similarly, Schwartz and Simon^15^ evaluated sleep extension in twelve tennis players and found improved serving accuracy. Swinbourne et al^17^ found 25 rugby players had improved total sleep time, sleep quality, reaction time, and beneficial changes in stress hormone expression after sleep extension. In all, these studies suggest that extending athletes’ total sleep time correlates with improvements in athletic performance.

Improving sleep with sleep extension appears to be one of the answers to improved athletic performance. However, an important part of such an intervention is its feasibility. College student-athletes are constantly juggling the demands of academics with training and conditioning, competitions, travel, and social lives. This leaves little time to prioritize and obtain the recommended hours of sleep. While studies have shown that sleep extension improves athletic performance, little has been done to address sleep barriers or provide approaches to increasing sleep.

Another concept to consider is sleep consistency, which involves the regularity of sleep-wake times and day-to-day variability.^3^ In the general population, consistent sleep patterns have been associated with improved sleep quality, mood, and cognitive function.^16^ Irregular sleep patterns have also been suggested as a novel risk factor for cardiovascular disease independent of more traditional risk factors such as cigarette smoking, sedentary lifestyle, elevated body mass index, diabetes, elevated systolic blood pressure, and high cholesterol.^5^ In the college population, improved sleep consistency and sleep duration was associated with better academic performance.^12^ Less is known about the impact of sleep consistency on collegiate athletes.

This study seeks to investigate the feasibility of a sleep extension intervention in NCAA Division I athletes across multiple sports. It was hypothesized that both male and female athletes in this population would have difficulty adhering to sleep extension.

## Methods

### Participants

A total of 50 Division I athletes between the ages of 18 years and 22 years from a single institution agreed to participate in this study. Subjects included 24 members of the men’s football team, 14 members of the women’s soccer team, 6 members of the men’s soccer team, and 6 members of the men’s baseball team. Overall, there were 14 female participants and 36 male participants. A summary of subject demographics is presented in Table 1. Written and informed consent were obtained from all athletes, and the study was approved by the Institutional Review Board.

**Table 1.** Participant demographics

Sport	Sex	Age, years	Subjects, N
Football	Male	18-22	24
Soccer	Female	18-22	14
Soccer	Male	18-22	6
Baseball	Male	18-22	6

### Experimental Design

The study occurred between February 2022 and July 2023 over a 4-week period during the academic school year but in the off-season of each respective sport and did not conflict with end-of-year school examinations or scheduled holiday breaks. Subjects wore a validated sleep monitoring device (WHOOP 4.0 band) for the entirety of the study.^2,9,10^ During the first 2 weeks of the study, subjects were instructed to sleep as they normally do. This served as their baseline sleep data. This was followed by a 2-week sleep extension intervention, where athletes were instructed to extend their nighttime sleep to 10 hours each night. Student athletes were reminded daily to extend their sleep to the desired 10 hours with in-person reminders by student athletic trainers and with online reminders by TeamWorks - a group messaging application.

### Objective Sleep Data

Sleep data were collected via the WHOOP 4.0 band - a wearable sleep tracker. The WHOOP band has been validated against polysomnography for sleep state categorization and biometric data.^9,10^ Recorded sleep data included total sleep time (TST), light sleep, rapid eye movement (REM) sleep, slow-wave sleep, sleep efficiency, and sleep consistency. Naps were purposefully not included in total sleep time. Including naps mixes 2 physiologically and behaviorally distinct constructs, which can obscure effects of an overnight intervention targeting consolidated sleep. Sleep efficiency is defined as the ratio between the total hours asleep compared with the total time in bed, with a higher score representing fewer night-time awakenings. Sleep consistency was calculated using a proprietary metric developed by WHOOP, Inc, which was adapted from the sleep regularity index (SRI) described by Phillips et al.^13^ Unlike the SRI, which compares sleep states 24 hours apart, the WHOOP algorithm assesses sleep consistency over a 4-day period at 24-hour intervals, assigning lower weights to more distant comparisons, and calculates scores on a 0% to 100% scale, where higher scores indicate lower variability in sleep timing.

### Data Analysis

Consistent with previous sleep extension research, the averages of daily measurements taken during baseline and sleep extension for each person were calculated to represent their baseline and intervention values, respectively. Participants were excluded from data analysis if they had <7 days of trackable sleep data over either of the 2-week data collection phases to ensure reliability of individual-level sleep estimates, as previous work demonstrates that fewer nights yield unstable measures of sleep duration and variability. To ensure data quality, outlier detection and removal were performed using Tukey’s rule. All extreme outliers were removed on an individual daily level. Statistical analysis was performed using the R programming language (Version 4.2.1) using the package “rstatix.”^6^

Objective sleep data were checked for normal data distribution with the Shapiro-Wilk test. If there was a normal distribution, a paired samples *t* test was used to compare data sets. If data had a non-normal distribution, a Wilcoxon signed rank test was used. Statistical significance level was set at an alpha level of 0.05. Due to the exploratory nature of our study, no adjustments were made for multiple hypothesis testing.

Sample size varied slightly by outcome due to differing data completeness requirements for specific sleep metrics. Metrics assessing sleep consistency required a greater number of valid and temporally distributed observations and were calculated only for participants meeting these criteria.

## Results

### Sleep Metrics

The average period of data collected for the 50 study participants during each phase of the study was 11.3 days during the baseline phase and 11.5 days during the sleep extension (intervention) phase (Figure 1 and Table 2). The baseline average TST was 7.63 hours (7 hours and 38 minutes, SD 0.88, N = 49), with no statistically significant change during the intervention phase (7.63 ± 0.88 hours vs 7.50 ± 0.86 hours; *P* = 0.27). The baseline sleep consistency was 60.09% (SD 12.6%, N = 46), which improved to 64.42% (SD 12.4%) during the intervention phase (p = 0.01). There were no statistically significant changes in any of the subcomponents of sleep architecture (time spent in light sleep (3.71 vs 3.80 hours; *P* = 0.36), REM sleep (1.54 vs 1.60 hours; *P* = 0.20), and slow-wave sleep (1.31 vs 1.31 hours; *P* = 0.98) cycles) or in sleep efficiency (0.9 vs 0.9 hours; *P* = 0.89) between baseline and sleep extension phases.

**Figure 1.**
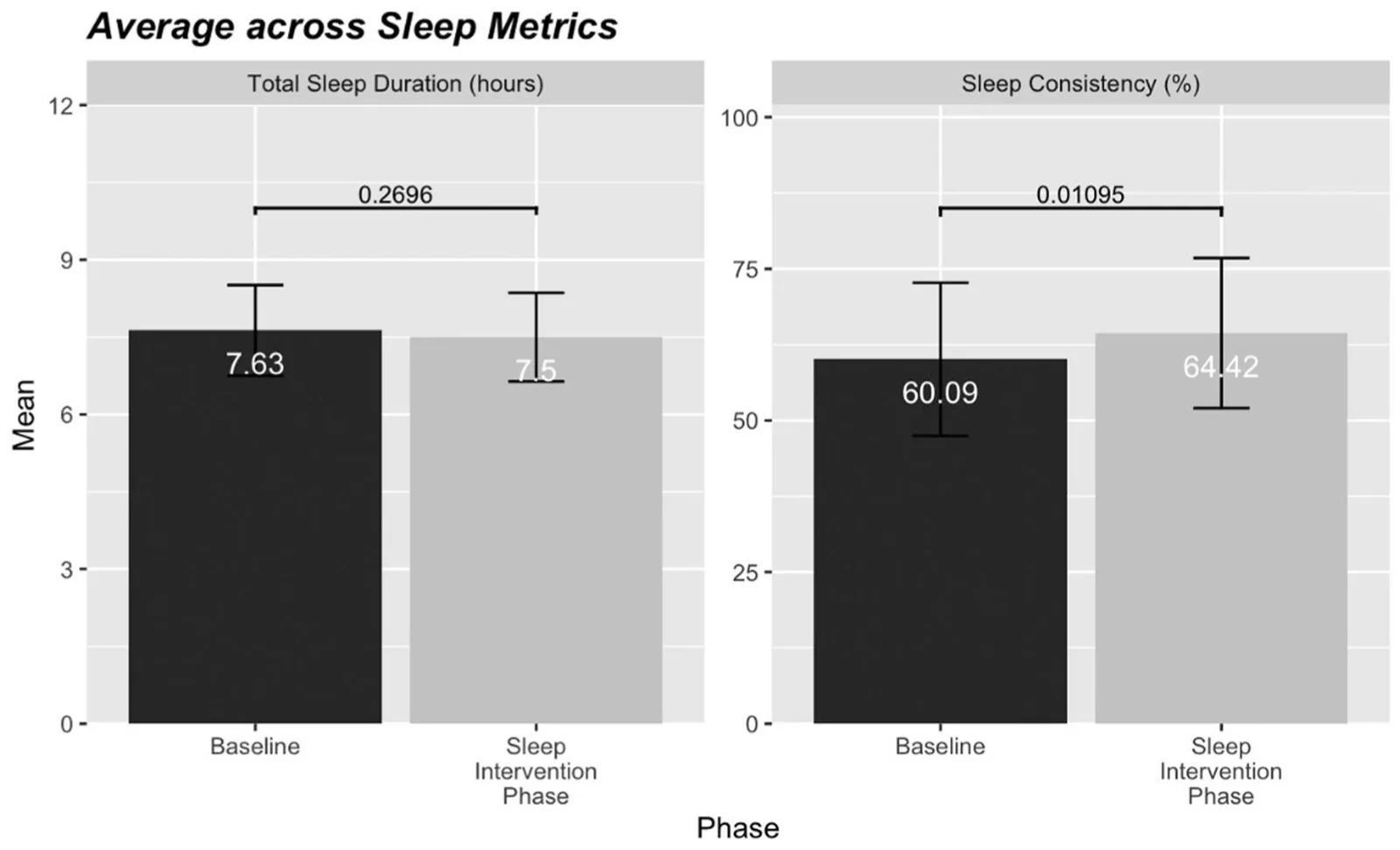
Sleep duration and consistency.

**Table 2.** Baseline versus sleep extension phase comparison

Metric	Sample size, N	Baseline	Sleep extension phase	*P* value	Statistical test
Sleep consistency, %	46	60.09 ± 12.6	64.42 ± 12.4	0.01	Paired *t* test
TST, hours	49	7.625 ± 0.879	7.504 ± 0.859	0.27	Wilcoxon signed rank test
Sleep duration, hours	49	6.763 ± 0.857	6.688 ± 0.785	0.40	Paired *t* test
Slow wave sleep duration, hours	50	1.31 ± 0.307	1.312 ± 0.327	0.98	Paired *t* test
REM sleep duration, hours	50	1.537 ± 0.54	1.603 ± 0.588	0.20	Wilcoxon signed rank test
Light sleep duration, hours	50	3.714 ± 1.045	3.798 ± 0.88	0.36	Wilcoxon signed rank test
Sleep efficiency	50	0.902 ± 0.037	0.902 ± 0.036	0.90	Paired *t* test

Values are mean ± SD unless otherwise stated. REM, rapid eye movement; TST, total sleep time.

### Sleep Metrics by Team

#### Women’s Soccer (N = 14)

The average period of data collection for the 14 study participants during each phase of the study was 11.0 days during the baseline phase and 10.7 days during the sleep extension phase. The baseline average TST was 7.88 hours (7 hours and 53 minutes, SD = 0.59, N = 13), with no statistically significant change during the intervention phase (7.88 ± 0.59 hours vs 7.79 ± 0.60 hours; *P* = 0.74). The baseline sleep consistency was 58.26% (SD = 14.93%, N=12), which improved to 69.43% (SD = 11.04%) during the intervention phase (*P* = 0.01). There were no statistically significant changes in any of the subcomponents of sleep architecture (time spent in light sleep (3.30 vs 3.64 hours; *P* = 0.14), REM sleep (1.80 vs 1.88 hours; *P* = 0.58), and slow-wave sleep (1.44 vs 1.49 hours; *P* = 0.48) cycles), or in sleep efficiency (*P* = 0.54) between baseline and sleep extension phases.

#### Men’s Soccer (N = 6)

The average period of data collection for the 6 study participants during each phase of the study was 12.5 days during the baseline phase and 9.5 days during the sleep extension phase. The baseline average TST was 8.07 hours (8 hours and 4 minutes, SD = 1.10, N = 6), with no statistically significant change during the intervention phase (8.07 ± 1.097 hours vs 8.05 ± 0.65 hours; *P* = 0.44). The baseline sleep consistency was 63.34% (SD = 7.12%, N = 6), with no statistically significant change during the intervention phase (63.34 ± 7.12 vs 69.35 ± 14.01%; *P* = 0.38). There were no statistically significant changes in any of the subcomponents of sleep architecture (time spent in light sleep (3.59 vs 3.65 hours; *P* = 0.55), REM sleep (1.51 vs 1.76 hours; *P* = 0.19), slow-wave sleep (1.48 vs 1.49 hours; *P* = 0.90) cycles), or in sleep efficiency (*P* = 0.44) between baseline and sleep extension phases.

#### Men’s Baseball (N = 6)

The average period of data collection for the 6 study participants during each phase of the study was 14.0 days during the baseline phase and 14.5 days during the sleep extension phase. The baseline average TST was 7.96 hours (7 hours and 58 minutes, SD = 0.42, N = 6), with no statistically significant change during the intervention phase (7.96 ± 0.42 hours vs 7.92 ± 0.52 hours; *P* = 0.80). The baseline sleep consistency was 70.74% (SD = 7.81%, N = 6), which improved to 74.86 ± 7.93% during the intervention phase (*P* = 0.001). There were no statistically significant changes in any of the subcomponents of sleep architecture (time spent in light sleep (4.14 vs 4.02 hours; *P* = 0.63), REM sleep (1.69 versus 1.71 hours; *P* = 0.79), slow-wave sleep (1.39 vs 1.40 hours; *P* = 0.88) cycles) or in sleep efficiency (*P* = 0.69) between baseline and sleep extension phases.

#### Men’s Football (N = 24)

The average period of data collection for the 24 study participants during each phase of the study was 10.1 days during the baseline phase and 11.7 days during the sleep extension phase. The baseline average TST was 7.30 hours (7 hours and 18 minutes, SD = 0.95, N = 24), with no statistically significant change during the intervention phase (7.30 ± 0.95 hours vs 7.11 ± 0.93 hours; *P* = 0.16). The baseline sleep consistency was 57.29% (S = 12.34%, N = 22), with no statistically significant change during the intervention phase (57.29 ± 12.34 vs 57.50 ± 10.00%, *P* = 0.91). There were no statistically significant changes in any of the subcomponents of sleep architecture (time spent in light sleep (3.88 vs 3.87 hours; *P* = 0.95), REM sleep (1.35 vs 1.38 hours; *P* = 0.65), slow-wave sleep (1.18 vs 1.14 hours; *P* = 0.43) cycles) or in sleep efficiency (*P* = 0.73) between baseline and sleep extension phases.

## Discussion

This study investigated the practicality of implementing a sleep extension protocol in men and women NCAA Division I athletes across multiple sports. Throughout the sleep extension intervention period, our athletes were not able to increase their total sleep time despite daily reminders, which differs from previously published studies. Specifically, there were no significant changes to TST between the baseline and the intervention phases, and no statistically significant changes in any other components of sleep architecture (time spent in light sleep, REM sleep, and slow-wave sleep cycles). Our cohort’s inability to extend their sleep despite continuous reminders raises awareness to the potential challenges of utilizing sleep extension in college student athletes. We suspect there are several contributing factors as to why sleep extension was not successful.

First, college athletes face numerous competing demands while balancing their academic, athletic, and social lives which impact their sleep. A survey of 409 student-athletes across 9 universities found student-athletes spent 50 hours per week on athletics, with additional time spent on other voluntary athletic activities, receiving treatment for injuries, and traveling for competitions.^1^ A large majority of the surveyed athletes (71%) also identified sleep as the single item their athletic time commitments prevented them from doing.^1^ Second, the rigorous training schedules of college athletes often include early morning or late evening sessions, which can conflict with regular sleep routines. Third, although our cohort was evaluated during the off-season, other considerations for in-season athletes include frequent travel for competitions, particularly across different time zones, which can also be disruptive to their sleep patterns. On top of their athletic pursuits, balancing a full-time academic workload with other social responsibilities further constrain their time and make it difficult to prioritize sleep. These factors can lead to physical exhaustion from training, and mental stress, which can adversely affect sleep quality. Finally, other considerations that may impact sleep include athlete living environments, such as noisy dormitories or shared living spaces and apartments, which may not be conducive to optimal sleep hygiene.

While there are challenges to using sleep extension in many college athletes, it is notable that our cohort had significant improvements in sleep consistency throughout the intervention phase. This finding is particularly important since collegiate athletes have been previously found to have poorer sleep consistency compared with the general population.^4^ The improvement in sleep consistency during the intervention phase represents a positive outcome and suggests that, even within the time constraints of a demanding schedule, collegiate student athletes can attain uniform sleep patterns when they are guided to prioritize sleep. This also raises awareness towards taking a more holistic approach to athlete wellbeing in collegiate sports programs that considers the balance between athletic commitments, academic responsibilities, and health needs, which includes sleep. Among the many strategies to improve sleep, sleep consistency may be a more achievable approach for student-athletes to incorporate. Further research is warranted to explore the potential impacts of sleep consistency on athletic and academic performance in collegiate athletes.

There are several limitations to our study. First, our study monitored sleep using a validated wearable device due to its ease of use and to promote compliance, but polysomnography remains the gold standard. Second, exclusion of participants with insufficient data may introduce selection bias if missingness was related to sleep behavior or external factors (e.g., travel, illness). In addition, missing data may not have been completely at random, which could influence estimates of sleep duration and consistency. Third, we acknowledge the potential impact of the Hawthorne effect where subjects may alter their behavior as a result of knowingly being observed. In our study, athletes had frequent reminders about extending their sleep which may have resulted in an inaccurate representation of their natural sleep pattern and may have contributed to improved sleep consistency we observed. Employing methods that minimize the impact of observation on participant behavior could yield more accurate representations of their natural sleep patterns.

An additional limitation of this study relates to our inability to accurately quantify total time in bed during the sleep extension period. While athletes were instructed to extend their nighttime sleep opportunity to 10 hours, the WHOOP platform is not validated to measure *time in bed*, but rather sleep staging once sleep onset has occurred. Estimating time in bed using actigraphy and heartrate data alone presents significant challenges, as activities performed while awake in bed can produce highly variable physiologic signals. For example, activities such as reading or meditation may closely resemble sleep patterns, whereas watching emotionally arousing media or engaging in sexual activity may result in elevated heartrate and variable movement despite the athlete being in bed. As such, inferring time in bed from wearable data risks substantial misclassification. While nightly self-reported time in bed could have been collected, previous literature demonstrates variable accuracy of self-reported sleep behaviors, particularly in athletic populations. Consequently, we elected not to provide speculative estimates of time in bed. This limitation prevents us from distinguishing whether the lack of increased total sleep time reflects difficulty initiating or maintaining sleep versus an inability to allocate sufficient time in bed, an important distinction with implications for sleep education, intervention design, and feasibility interpretation. Future studies incorporating validated time-in-bed measures or mixed-methods approaches may help better clarify these mechanisms.

Our study population was also limited to Division I collegiate athletes and had a smaller sample size of female athletes. Additional studies are needed on other athletic populations at varying levels of competition with larger cohorts of female athletes. We intentionally performed our study during the off-season to avoid possible confounders and logistical challenges related to athlete travel across multiple time zones for competition but also recognize the importance of understanding sleep behaviors for in-season athletes. Finally, due to the exploratory nature of our study, which involved repeated daily measures of objective sleep and recovery metrics, we did not designate a primary outcome a priori and decided not to adjust for multiple hypothesis testing. This decision was made to avoid missing potentially meaningful associations that may warrant further exploration that may be rejected with stringent adjustment for multiple comparisons, therefore increasing the risk of type 2 errors. Future research with larger sample sizes is warranted to expand upon the findings suggested in our study.

## Conclusion

Sleep extension may be challenging to implement in NCAA Division I collegiate athletes due to a variety of competing demands but may result in improved sleep consistency patterns. Future research should focus on the specific barriers to achieving sleep extension in the student-athlete population, as well as the potential role of sleep consistency on athletic performance and athlete well-being.

## Supplemental Material

sj-docx-1-sph-10.1177_19417381261496986 – Supplemental material for Enhancing Sleep in NCAA Division I Athletes: A Study of Sleep Extension Feasibility Supplemental material, sj-docx-1-sph-10.1177_19417381261496986 for Enhancing Sleep in NCAA Division I Athletes: A Study of Sleep Extension Feasibility by Joshua T. Goldman, Calvin Duffaut, Nicolas Hatamiya, Basil Ike, Brian Donohoe, Nelson F. Boland, Jeremy Vail, Jeongeun Kim and Kristen E. Holmes in Sports Health
