# Supplementary material for: Enhancing Sleep in NCAA Division I Athletes: A Study of Sleep Extension Feasibility
Source: Sports Health. 2026 Oct 28:19417381261496986. Online ahead of print. doi: 10.1177/19417381261496986 (PMC13716884; doi:10.1177/19417381261496986)
Supplement: Supplementary file 1 [file sj-docx-1-sph-10.1177_19417381261496986.docx]

**UNIVERSITY OF CALIFORNIA, LOS ANGELES	UCLA**

| BERKELEY • DAVIS • IRVINE • LOS ANGELES • RIVERSIDE • SAN DIEGO • SAN FRANCISCO |  | SANTA BARBARA • SANTA CRUZ |
| --- | --- | --- |

JOSHUA T. GOLDMAN, MD, MBA	DIVISION OF SPORTS MEDICINE

HEALTH SCIENCES ASSOCIATE CLINICAL PROFESSOR	DEPARTMENT OF FAMILY MEDICINE

jgoldman@mednet.ucla.edu	DEPARTMENT OF ORTHOPEDIC SURGERY

100 MEDICAL PLAZA, SUITE170

LOS ANGELES, CA 90049

(424) 259-9832	FAX (424) 259-6561

December 23, 2025

Edward M. Wojtys, MD
University of Michigan

24 Frank Lloyd Wright Dr Ste 1000

Ann Arbor, MI 48105

Dear Dr. Wojtys:

I am pleased to resubmit an original research article entitled “Enhancing Sleep in NCAA Division I Athletes:

A Study of Sleep Extension Feasibility” from our research group at UCLA for consideration for publication in the *Sports Health*.

We are grateful for the thoughtful and constructive feedback provided by the reviewers and have revised the manuscript accordingly. In particular, we have expanded the Discussion to explicitly address the distinction between time in bed versus total sleep time, clarifying important methodological limitations related to wearable sleep technology and the implications this distinction has for interpreting feasibility, sleep education, and future intervention design. We believe these revisions meaningfully strengthen the manuscript’s clarity, scientific rigor, and clinical relevance.

In this study, we demonstrate that sleep extension may be unfeasible for many collegiate athletes, even when implemented under optimized conditions, including the off-season, remote from academic examinations, and with frequent reminders and oversight. To our knowledge, this is the first study to evaluate the feasibility of a sleep extension intervention in NCAA Division I athletes using modern, validated wearable sleep monitoring technology. While prior foundational work—most notably by Dr. Cheri Mah and colleagues—has shown performance benefits of sleep extension, those studies relied on older monitoring technologies and small, highly controlled cohorts. Our findings suggest that, despite well-documented performance benefits, sleep extension may represent an impractical intervention for many collegiate student-athletes in real-world settings.

We believe this manuscript is well aligned with the mission and readership of Sports Health due to its direct clinical relevance to sports medicine physicians, athletic trainers, and performance staff who routinely counsel athletes on sleep and recovery strategies. Sports Health has a strong history of publishing impactful work in this area, including prior studies on sleep quality, variability, and mental health in collegiate athletes. We are enthusiastic about the opportunity to contribute to this literature and would be honored to publish our work in Sports Health.

This manuscript has not been published previously and is not under consideration elsewhere. The authors have no conflicts of interest to disclose.

Thank you very much for your time and consideration. We appreciate the opportunity to revise and resubmit our work and look forward to your decision.

Sincerely,

**Joshua T. Goldman, MD, MBA**

Associate Clinical Professor, UCLA Departments of Family Medicine & Orthopedic Surgery

Associate Director, Center for Sports Medicine, Orthopedic Institute for Children

Associate Director, UCLA Steve Tisch BrainSPORT Program

Program Director, UCLA Sports Medicine Fellowship

Team Physician, Los Angeles Chargers & UCLA Department of Intercollegiate Athletics
